# Prevalence of Joint Hypermobility and Patterns of Articular Manifestations in Patients with Inflammatory Bowel Disease

**DOI:** 10.1155/2009/924138

**Published:** 2010-02-11

**Authors:** P. Vounotrypidis, E. Efremidou, P. Zezos, M. Pitiakoudis, E. Maltezos, N. Lyratzopoulos, G. Kouklakis

**Affiliations:** ^1^Endoscopy Unit & Inflammatory Bowel Diseases Unit, University General Hospital of Alexandroupolis, Democritus University of Thrace, Alexandroupolis 68100, Greece; ^2^1st Surgical Department, University General Hospital of Alexandroupolis, Democritus University of Thrace, Dragana, Alexandroupolis 68100, Greece; ^3^2nd Surgical Department, University General Hospital of Alexandroupolis, Democritus University of Thrace, Alexandroupolis 68100, Greece; ^4^2nd Department of Internal Medicine, University General Hospital of Alexandroupolis, Democritus University of Thrace, Alexandroupolis 68100, Greece

## Abstract

*Objective*. The objective is the investigation of Joint Hypermobility (JH) and the Hypermobility Syndrome (HMS) in patients with inflammatory bowel disease (IBD). *Methods*. We examined 83 patients with IBD and 67 healthy individuals for the presence of JH. Patients were excluded if they were under 18 or over 50 years of age and if they had other conditions which affect joint mobility. The x^2^ and the Fisher exact test were used appropriately between study groups. Odds ratios (ORs) for the risk of JH and HMS in IBD groups were calculated. *Results*. A total of 150 individuals (83 IBD patients and 67 healthy controls) participated in the study. 69 IBD patients, 41 with Crohn's Disease (CD) and 28 with ulcerative colitis (UC), were finally eligible. JH was detected in 29 CD patients (70.7%), in 10 UC patients (35.7%), and in 17 healthy control subjects (25.4%). Significant difference was detected on JH in CD patients as compared to UC patients (*P* = .0063) and controls (*P* < .0001). The estimated OR for JH was 7.108 (95% CI: 2.98–16.95) in CD and 1.634 (95% CI: 0.63–4.22) in UC patients. HMS was detected in 5 (12.2%) CD and in 1 (3.57%) UC patients. The OR for HMS in CD was 3.75 (95% CI: 0.41–34.007), while 7 (17.1%) CD patients had overlapping symptoms for both HMS and early spondylarthropathy. *Conclusions*. JH and the HMS are common in CD patients, thus articular manifestations should be carefully interpreted. This implies an involvement of collagen varieties in the pathogenesis of IBD.

## 1. Introduction

Joint hypermobility (JH) is a condition characterized by joint laxity and an increased range of joint motion. In general population is deemed as the upper range of a normal distribution while it is also a feature of genetic disorders such as Marfan's, Ehlers-Danlos syndromes and Osteogenesis Imperfecta. There are marked ethnic, age and gender differences in articular mobility. Beighton's scoring method (Table 1) incorporates nine sites to evaluate articular mobility and it is widely accepted and easily applicable in the clinical practice. In some individuals JH gives rise to musculoskeletal problems the so called hypermobility syndrome (HMS).

Following the first association between joint hypermobility and musculoskeletal complaints ninety years ago and the definite description of HMS, in recent years many reports have found publicity on various extra-articular manifestations of JH and its association to HMS. There are no published studies of benign JH in patients with inflammatory bowel disease (IBD). Trigger of the study was the observation that patients with IBD appeared to have hypermobile joints. The aim of this study was to formally evaluate the incidence of joint hypermobility and the hypermobility syndrome in patients with Crohn's disease (CD) and ulcerative colitis (UC).

There are no published studies of benign joint hypermobility in patients with inflammatory bowel disease (IBD). Trigger of the study was an observation, in a setting of a combined gastroenterology and rheumatology outpatient clinic that patients with IBD appeared to have hypermobile joints. The aim of this study was to formally evaluate the incidence of joint hypermobility and the hypermobility syndrome in patients with Crohn's disease (CD) and ulcerative colitis (UC).

## 2. Patients and Methods

In the study enrolled 83 patients with IBD who attended consecutively, between January 2007 and September 2008, the Inflammatory Bowel Diseases outpatient clinic at the University Hospital of Alexandroupolis. For the prospective cohort, patient history was obtained, and a physical examination was performed in all cases by the same rheumatologist (PV). We recorded the familial history regarding the presence of IBD, ankylosing spondylitis, psoriasis and features of JH in the first degree relatives of patients.

Initially the rheumatologist was not blinded for the diagnosis of IBD. This was the period in which the observation of the increased incidence of joint hypermobility in patients with CD was made. Twenty eight patients (33.7%) were examined in non blinded fashion.

The joint laxity was evaluated using Beighton's scoring method. Meanwhile the 1998 Brighton's criteria were used to identify patients with HMS (Table 2) [1]. 

Radiological evidence of sacroiliitis and the presence of synovitis established the diagnosis of seronegative enteropathic ankylosing spondylitis-spondyloarthritis (SpA) according to the European Spondylarthropathy Study Group (ESSG) criteria.

The diagnosis of Crohn's disease and ulcerative colitis had been established by the standard clinical, endoscopic and histologic criteria.

Patients were excluded if they met any of the below described criteria: (a) patients younger than 18 or older than 50 years, in order to minimize the age related effect on joint laxity, (b) patients with a history of SpA which has affected joints that are calculated in the scoring method, (c) patients with any other pathological hypermobility condition (e.g., acromegaly, hyperparathyroidism, chronic alcoholism, rheumatic fever) [2]. 

The duration of oral steroid treatment was recorded for both CD and UC patients for the period of the previous year before their entry to the study. We also performed a subgroup analysis of the concomitant treatment in both CD and UC patients in relation to joint hypermobility.

Another group of 96 employees of the hospital agreed to participate and to serve as a control group. They were screened and we excluded those with chronic diseases or previously treated with steroids. The remaining controls were age and sex matched with patients. 

All patients and control subjects gave their informed consent to participate in the study. The research protocol was approved by the Hospital's Scientific Committee.

Statistical analyses were performed using the SPSS for Windows package (version 11.0, SPSS, Chicago, IL). Data are presented as medians along with minimum and maximum values. The Chi square test and the Fisher's exact test were used when appropriate to compare the proportions of joint hypermobility between the study groups. Odds ratios (OR) and the 95% CI were calculated as well as the Relative Risk (RR) of JH in patients with CD and UC, compared to healthy controls. The level of significance was set at 0.05.

## 3. Results

A total of 150 Greek Caucasians (83 IBD patients and 67 healthy individuals) participated in the study. Sixty nine out of 83 IBD patients (41 with CD and 28 with UC) were finally eligible. Ten IBD patients were excluded due to age restrictions. Three CD and one UC patients were excluded due to ankylosing spondylitis (AS). Oral steroid treatment for the past year was recorded in both groups in order to detect any differences in cumulative steroid treatment which may also interfere to the manifestation of joint laxity. 

Overall 67 healthy controls were possible to be age and sex matched with patients to avoid selection bias and they finally included in the study. Demographical data of IBD patients and controls are shown in Table 3. 

Joint hypermobility was detected in 29 out of 41 CD patients (70.7%), in 10 out of 28 UC patients (35.7%) and in 17 out of 67 healthy control subjects (25.4%). There was a significant difference in the proportion of joint hypermobility in CD patients as compared to UC patients (Fisher's exact two-tailed test: *P* = .0063) and healthy controls (Fisher's exact two-tailed test: *P* < .0001). No differences were found in the proportion of joint hypermobility between UC patients and healthy controls (*P* = .3275).

The estimated odds ratios (OR) for joint hypermobility were 7.108 (95% CI: 2.98–16.95) in CD patients and 1.634 (95% CI: 0.63–4.22) in UC patients, as compared to healthy controls. 

The relative risk for the coexistence of JH and CD is RR: 3.257 (95% CI 1.87–5.67), while the coexistence of JH and UC is RR: 1.399 (95% CI: 0.74–2.63). According to these data it is two times more likely that the underlying IBD in a patient with joint hypermobility is CD rather than UC [RR: 1.859 (95% CI: 1.15–2.99)].

Analyzing data regarding their treatment we found that seventeen patients with CD (41.4%) and eleven patients with UC (39.3%) were on steroids during the previous year for a median period of 3.17 months (range: 2–12) and 2.78 months [range: 2–12] respectively. The JH to Non-JH ratio in CD patients who were treated with steroids was 10 : 7 while in UC patients was 4 : 7. Interestingly, these data suggest that there is no apparent effect of steroid treatment in our cohort. 

Concomitant immunomodulating and antibiotic therapy in this cohort was also recorded (Table 4).

Although there are among CD patients treated with anti-TNF alpha agents (Infliximab or Adalimumab) an increased number of patients fulfilling our criteria for JH the difference is not statistically significant compared to those CD patients who did not received the same treatment (Fisher's exact two-tailed test *P* = .452). Borderline insignificance on JH was detected between CD and UC patients who received aminosalycilate or azathioprine (Fisher's exact two-tailed test *P* = .079 and *P* = .056 resp.). 

In order to investigate the clinical relevance of JH we analyzed the patterns of articular manifestations in our cohort of IBD patients. A significant proportion of articular manifestations attributed to HMS were found among CD patients. HMS was detected in 5 (12.2%) CD and in 1 (3.57%) UC patients [OR: 3.75 (95% CI: 0.41–34.007). Additionally, there were 7 CD patients (17.1%), who had overlapping symptoms for both HMS (Brighton's major and minor criteria 1 and 2), and early spondylarthropathy (i.e., artharalgias, enthesitis and longstanding inflammatory back pain without radiographic evidence of permanent lesions) (Table 3). This overlap was not detected in patients with UC who seem to have more overt symptoms and well defined articular manifestations such as degenerative spondylarthritis and osteoarthritis or definite and radiographically evidenced SpA.

### 3.1. Discussion

In this study we demonstrate an increased articular mobility in CD patients compared to UC patients and healthy control subjects, while there were no differences between UC patients and healthy controls. The estimated Odds ratio for joint hypermobility in CD patients was 7.108 (95% CI: 2.98–16.95). To the best of our knowledge there is no other study to correlate joint hypermobility with IBD.

The prevalence of JH is increased in childhood and decreases with age in relation with other factors such as occupational and manual activities, thus we used an observational window consisted of ages between 18 and 50 years-old to eliminate any age related effect on joint mobility. We studied a homogenous Caucasian Greek population, therefore due to the ethnic differences in articular mobility [2–10], the identification in other ethnic groups of a connection between IBD, especially CD and joint hypermobility could give an indirect evidence of the importance of collagen or connective tissue matrix involvement as part of the pathogenesis of the intestinal disease. 

There are no published data of JH in the Greek population, although a large study in Greece has shown an overall 20% prevalence of low back pain, neck pain and soft tissue rheumatism (i.e., tendinitis, bursitis, etc.) [11]. Patients and controls in our cohort were enrolled from the grater region of Thrace and central and eastern Macedonia, which are accounted as the one forth of the Greek population so they may considered representative. The prevalence of JH in our controls is high (25.4%) and close to that reported in people of Eastern rather than those of Western populations [6, 10]. 

In our cohort both CD and UC patients were treated similarly in terms of oral steroids the year before their recruitment. We found that the majority of UC patients who were on steroid treatment had no features of JH, thus there is no apparent effect of steroids on articular mobility. In a subgroup analysis of the concomitant medications a significant proportion of CD patients who were treated with anti-TNF agents had Beighton scores suggesting JH but there was not statistical difference among them and the CD patients who were not on the specific treatment. 

Joint hypermobility was not influenced by the type of medication in both CD and UC patients. The apparent predominance of JH on CD patients who were treated with azathioprine or aminoalycilates is borderline insignificant compared to JH in UC patients (*P* = .079 and *P* = .056, resp.). A larger sample could possibly detect differences which rather enhance the high prevalence of JH in CD than the true effect of these medications on joint laxity. 

A rising question is whether JH is secondary to IBD. The intestinal inflammation does not seem to be the primary reason for secondary joint hypermobility in patients with CD, since the median disease duration was higher in UC, 5 years [0.1–20] versus 3 years [0.1–21] in CD.

It is of particular interest that a large number of patients with CD exhibit articular manifestations defined as HMS. This is reported for a first time and provides to the clinicians important information regarding the interpretation of musculoskeletal symptoms on patients with CD. Our study accomplish a recent observation that patients with JH exhibit unexplained gastrointestinal symptoms [12]. 

The incidence of SpAs including AS in our cohort is within the range of 2%–20% reported in the literature for CD patients (16.3%) and slightly higher in UC patients (23.5%). [13] On the other hand, the hypermobility joint syndrome has a 12.2% prevalence in CD versus 3.5% in UC patients. Interestingly a substantial proportion (17.7%) of patients with CD had overlapping joint symptoms, which were arthralgia and/or inflammatory spinal pain and this could indicate either premature SpA (i.e. without radiographic evidence of sacroiliitis) or HMS. Thus, articular manifestations in a patient with CD should be interpreted carefully in the direction of spondylarthropathy or HMS.

## 4. Conclusions

Joint hypermobility and the hypermobility syndrome are common in Crohn's disease patients in the Greek Caucasian population while there is no difference on joint hypermobility in patients with ulcerative colitis compared to the healthy control group. It is two times more likely that the underlying IBD of a patient who exhibits joint hypermobility is CD rather than UC. The constellation of articular symptoms in CD patients does not always suggest an enteropathic spondylarthropathy but the coexistent joint hypermobility. The high incidence of this particular phenotype of JH on CD patients could support the hypothesis that collagen varieties may attribute to the pathogenesis of CD.

## Figures and Tables

**Table 1:** Beighton's joint hypermobility score.

The ability to	Right	Left
(1) Passively dorsiflex the fifth metacarpeophalangeal joint to ≥90°	1	1
(2) Oppose the thumb to the volar aspect of the ipsilateral forearm	1	1
(3) Hyperextend the elbow to ≥10°	1	1
(4) Hyperextend the knee to ≥10°	1	1
(5) Place hands flat on the floor without bending the knees	1	

Total possible score	9	

One point can be gained for each side for manoeuvres 1–4 so that the hypermobility score will have a maximum of 9 points if all are positive.

**Table 2:** Brighton's diagnostic criteria for hypermobility syndrome.

*Major Criteria*
Beighton score 4/9 or greater (either currently or historically)
Arthralgia for longer than 3 months in 4 or more joints
*Minor criteria*
Beighton score of 1–3/9 (0–3 if aged 50+)
Arthralgia in 1–3 joints or back pain or spondylosis, spondylolisthesis
Dislocation in more than 1 joint, or in 1 joint or more on more than 1 occasion
Three or more soft tissue lesions (e.g., epicondylitis, tenosynovitis, bursitis)
Marfanoid habitus (tall, slim, span>height, upper segment : lower segment ratio <0.89, Arachodactyly)
Skin striae, hyperextensibility, thin skin or abnormal scarring
Eye signs: drooping eyelids or myopia or antimongoloid slant
Varicose veins or hernia or uterine/rectal prolapse
Mitral valve prolapse (by echocardiography)

*HMS diagnosis requires*
Two major criteria *or *
One major + two minor criteria *or *
Four minor criteria *or *
Two minor criteria and unequivocally affected first-degree relative

HMS is excluded by the presence of marfan or Ehlers-Danlos Syndromes.

**Table 3:** Demographic and clinical data of IBD patients and controls.

	Crohn's disease	Ulcerative colitis	Controls
N	49	34	96

Number of participants	41	28	67
Sex (M/F)	22/19	15/13	38/29
Age (range)*	32 (18–50)	32.5 (18–49)	32 (18–50)
Disease duration in years (range)*	3 (0.1–21)	5 (0.1–20)	
Patients on oral steroids the past year (%)	17 (41.4)	11 (39.2)	
Joint Hypermobility (%)	29 (70.3)	10 (35.7)	17 (25.4)
Median Beighton Scores [range]			
In total population	4 [0–9]	3 [0–8]	2.5 [0–9]
In hypermobile subjects	5 [4–9]	5 [4–8]	5 [4–9]
Articular Manifestations (%)			
Spondylarthropathy (SpA)^†^	5 (12.2)	7 (25)	
Hypermobility Syndrome (HMS)	5 (12.2)	1 (3.57)	
Overlap symptoms (SpA & HMS)	7 (17.1)	0	
Non specific or Degenerative Symptoms	11 (26.8)	7 (25)	
Patients excluded from the study			
Age restriction	5	5	
Spondylarthropathies (AS)	3	1	

*Data are expressed as medians along with minimum and maximum values.

^†^Arthritis which does not influence the Beighton score counting.

**Table 4:** Medication of IBD patients in relation to Joint Hypermobility. A number of patients were on combination treatment (data not shown).

Treatment	Crohn's disease N	Ulcerative colitis N
	(JH/Non-JH)	(JH/Non-JH)
Steroids the last year	**17** (10/7)	**11** (3/8)
Aminosalycilates	**19** (12/7)	**20** (7/13)
Azathioprine	**14** (11/3)	**7** (2/5)
Anti-TNF	**12** (10/2)	**0**
Antibiotics	**5 **(3/2)	**2** (1/1)
Methotrexate	**1** (1/0)	**1** (0/1)
Mercaptopourin	**1** (0/1)	**1** (0/1)
